# Effect of caffeine ingestion on cycling performance: a systematic review and meta-analysis

**DOI:** 10.3389/fnut.2025.1745472

**Published:** 2026-01-12

**Authors:** Jinyan Wu, Kai Xu, Mingyue Yin, Xianming Ding, Tong Wang, Qiubo Zhang, Xiaowei Wu, Ningkun Xiao

**Affiliations:** 1 Chengdu Sport University, Chengdu, China; 2 School of Medical and Health Sciences, Centre for Human Performance, Edith Cowan University, Joondalup, WA, Australia; 3 School of Athletic Performance, Shanghai University of Sport, Shanghai, China; 4 Exercise and Nutrition Research Program, The Mary MacKillop Institute for Health Research, Australian Catholic University, Melbourne, VIC, Australia; 5 Jishou University, Jishou, China; 6 Sichuan Normal University, Chengdu, China; 7 Department of Immunochemistry, Institution of Chemical Engineering, Ural Federal University, Yekaterinburg, Russia; 8 Laboratory for Brain and Neurocognitive Development, Department of Psychology, Institution of Humanities, Ural Federal University, Yekaterinburg, Russia

**Keywords:** caffeine, cycling, exercise performance, meta-analysis, sports nutrition

## Abstract

This study aimed to determine the effects of acute caffeine ingestion on cycling performance through a systematic review and meta-analysis, while also exploring the moderating roles of caffeine dosage, training status, and athlete age. A comprehensive search was conducted across five databases, yielding 20 eligible studies with a total of 226 participants. A three-level mixed-effects model was applied to pool main effects on cycling time trial performance, mean power output, mean heart rate, and ratings of perceived exertion (RPE). Subgroup analyses and meta-regression were performed to examine potential moderators. Caffeine intake significantly reduced cycling completion time (SMD = −0.36, 95% CI: −0.57 to −0.15, *p* = 0.0017) and increased mean power output (SMD = 0.29, 95% CI: 0.05 to 0.52, *p* = 0.02), but had no significant effect on heart rate or RPE. Subgroup analysis indicated that a low dose of caffeine (≤3 mg/kg; SMD = −0.42) was more effective in reducing completion time compared with a higher dose (4–6 mg/kg; SMD = −0.34). Meta-regression further revealed a significant moderating effect of age on time trial performance (*β* = −0.0501; *p* = 0.02). Taken together, these findings suggest that ingesting caffeine approximately one hour before exercise can effectively enhance cycling performance, with low doses achieving improvements comparable to higher doses.

## 1 Introduction

In recent years, nutritional supplementation has been widely recognized as an important ergogenic aid to enhance athletic performance, delay fatigue, and improve metabolic regulation (1, 2). Among the numerous supplements, caffeine has become one of the most popular due to its accessibility and well-documented efficacy in both elite and recreational sports (3, 4). Acute caffeine ingestion exerts its ergogenic effects through multiple mechanisms, including antagonism of adenosine receptors, increased central nervous system excitability, enhanced muscle contractility, promotion of fat mobilization and utilization, and reduced perceived exertion during exercise (5–7). These mechanisms make caffeine particularly promising in endurance-based sports (8–10).

Cycling, due to its standardized format and strong experimental controllability, has been widely used as a model for evaluating the ergogenic effects of nutritional interventions, including caffeine (11–13). Although numerous studies have investigated the acute effects of caffeine on cycling performance (14–16), the findings remain inconsistent. Several studies reported that caffeine significantly reduced time trial completion time and improved power output (17–19), whereas others failed to observe significant effects and even noted large inter-individual variability in responses (20). A recent meta-analysis summarized the effects of caffeine ingestion on cycling time trials and demonstrated significant improvements in completion time and mean power output (21).

However, considering that the previous review included a limited number of studies, did not comprehensively cover endurance-related performance outcomes, and did not clearly evaluate the certainty of evidence using GRADE, it is necessary to conduct a more rigorous synthesis of the literature—one that incorporates a broader range of endurance performance indicators and employs more advanced analytical methods (three-level meta-analytic approach). Such an effort would allow for a more robust evaluation of the ergogenic effects of caffeine during cycling and enable further exploration of potential moderators, thereby providing stronger evidence to support nutritional strategies in athletic practice. Therefore, the present study aimed to systematically review and meta-analyze high-quality research on the acute effects of caffeine ingestion on cycling performance, with a focus on key outcomes including cycling completion time, power output, mean heart rate, and ratings of perceived exertion.

## 2 Materials and methods

### 2.1 Literature search

This systematic review and meta-analysis was conducted in accordance with the Preferred Reporting Items for Systematic Reviews and Meta-Analyses 2020 guidelines (22). The protocol was prospectively registered on the International Prospective Register of Systematic Reviews (PROSPERO; registration number: CRD420251016970). A comprehensive search was performed in five electronic databases—PubMed, Embase, Scopus, Cochrane Library, and Web of Science—from their inception to March 1, 2025. The search strategy was developed based on the PICOS framework (Population, Intervention, Comparator, Outcomes, Study design) (23). Details of the search terms are presented in Supplementary material A. Within each PICOS component, keywords were combined using the Boolean operator “OR,” and the components were linked using “AND” to execute the final search.

### 2.2 Study selection

One investigator (W. J. Y.) independently removed duplicate records using EndNote 21 software. Subsequently, two investigators (W. J. Y. and W. X. W) independently screened the titles and abstracts of the retrieved studies based on the inclusion and exclusion criteria. Full texts were reviewed when eligibility could not be determined from the title and abstract. Any disagreements were resolved through discussion, and a third investigator (D. X. M.) was consulted when necessary.

### 2.3 Inclusion and exclusion criteria

The inclusion and exclusion criteria were established according to the PICOS framework. Inclusion criteria: (1) Population: Healthy individuals, including well-trained and recreational cyclists. Well-trained athletes were defined as having ≥3 years of systematic training, cycling ≥4–5 times per week, with a total weekly training volume of ≥8–10 h. Recreational cyclists were defined as having some cycling experience but training ≤3 times per week, with a total weekly training volume <6 h, and lacking long-term systematic competitive training experience (24–26). (2) Intervention: Caffeine ingestion prior to formal exercise, Forms of ingestion include solutions, tablets, mouthwash, and capsules. (3) Comparator: The same protocol as the intervention group, except that participants ingested a placebo instead of caffeine. (4) Outcomes: At least one cycling performance-related outcome, such as completion time, mean power output, mean heart rate, or ratings of perceived exertion (RPE).(5) Study design: Randomized controlled or crossover trial. Exclusion criteria: (1) Studies involving participants with clinical conditions; (2) Animal studies; (3) Qualitative studies, systematic reviews, meta-analyses, study protocols, preprints, and conference abstracts not peer-reviewed. (4) Excluded all anaerobic maximal sprint tests (e.g., Wingate tests).

### 2.4 Data extraction

Two investigators (W. J. Y. and W. X. W) independently extracted data, including bibliographic details, participant characteristics, caffeine dosage, and primary outcome measures. A third investigator (D. X. M.) checked the extracted data, and any disagreements were resolved by a fourth investigator (X. N. K.) through arbitration. If essential data were missing, the corresponding authors were contacted. Studies with unavailable data were excluded from the analysis.

### 2.5 Risk of bias assessment

Two investigators (W. J. Y. and D. X. M) independently assessed the risk of bias for each included study using the Cochrane Risk of Bias 2 (RoB 2) tool (27), evaluating the randomization process, deviations from intended interventions, missing outcome data, outcome measurement, and selective reporting. Disagreements were first discussed and, if unresolved, adjudicated by a third investigator (X. N. K.).

### 2.6 Outcome measures

Two investigators (W. J. Y. and D. X. M) independently extracted data from each study and cross-verified the information for consistency. Extracted data included: (1) Study characteristics: Author(s), publication year, and country; (2) Participant characteristics: Sample size, sex distribution, mean age with standard deviation, health status (healthy adults), and training level; (3) Intervention details: Caffeine dosage (mg or mg/kg), timing of ingestion relative to exercise, administration form (capsule, beverage), and placebo control; (4) Study design features: Study type, blinding status, and type of cycling task; (5) Primary outcomes: Cycling completion time (seconds or minutes), mean power output (watts), mean heart rate (bpm), and RPE score. Given potential heterogeneity across studies regarding participant characteristics and caffeine dosage, subgroup analyses were pre-specified: (1) Caffeine dosage: Low dose (≤3 mg/kg) vs. moderate dose (3–6 mg/kg) (28, 29); (2) Training level: Well-trained vs. recreational cyclists (24–26).

### 2.7 Certainty of evidence

The certainty of evidence was assessed using the Grading of Recommendations Assessment, Development, and Evaluation (GRADE) system (30), which classifies evidence as high, moderate, low, or very low. Two investigators (W. J. Y. and D. X. M) independently performed the GRADE assessment.

### 2.8 Statistical analysis

All statistical analyses were conducted using R software (version 4.5.0), primarily relying on the metafor (31), clubSandwich (32), and puniform (33) packages for data processing and model construction. Based on the means, standard deviations, and sample sizes of the intervention and control groups, the standardized mean difference (SMD) was calculated as the effect size to evaluate the impact of acute caffeine ingestion on cycling performance. Effect sizes were interpreted as trivial (SMD < 0.20), small (0.20 ≤ SMD < 0.50), moderate (0.50 ≤ SMD < 0.80), or large (SMD ≥ 0.80) (34). To account for the nested structure of effect sizes within studies, a three-level random-effects model was constructed, with the study as level 3, individual effect sizes (id) as level 2, and residuals as level 1. The random-effects structure was specified as ~1|study/id (35, 36). Model parameters were estimated using the restricted maximum likelihood (REML) method (37), and robust variance estimation (RVE) was applied with the CR2 correction matrix from the clubSandwich package to adjust for small-sample bias, providing robust confidence intervals and significance tests (38, 39). Heterogeneity among studies was assessed using Cochran’s Q statistic, which evaluates the variability of the main effects across studies (40). I^2^ was also calculated to quantify heterogeneity, classified as low (I^2^ < 25%), moderate (25% ≤ I^2^ ≤ 50%), or high (I^2^ > 50%) (41). Statistical significance was set at *p* < 0.05. Effect size calculations were performed using the escalc function (31), and results were visualized with forest plots showing individual study effects and pooled estimates. Publication bias was assessed using funnel plots and Egger’s regression test (42, 43). To examine the robustness of pooled effect sizes, leave-one-out sensitivity analyses were conducted to evaluate the influence of individual studies (31). Considering potential variability in caffeine dosage, training status, and age among participants, subgroup analyses were performed by dosage and training level, and meta-regression analyses were conducted to explore the moderating effects of age on the intervention outcomes.

## 3 Results

### 3.1 Literature search results

**Figure 1:**
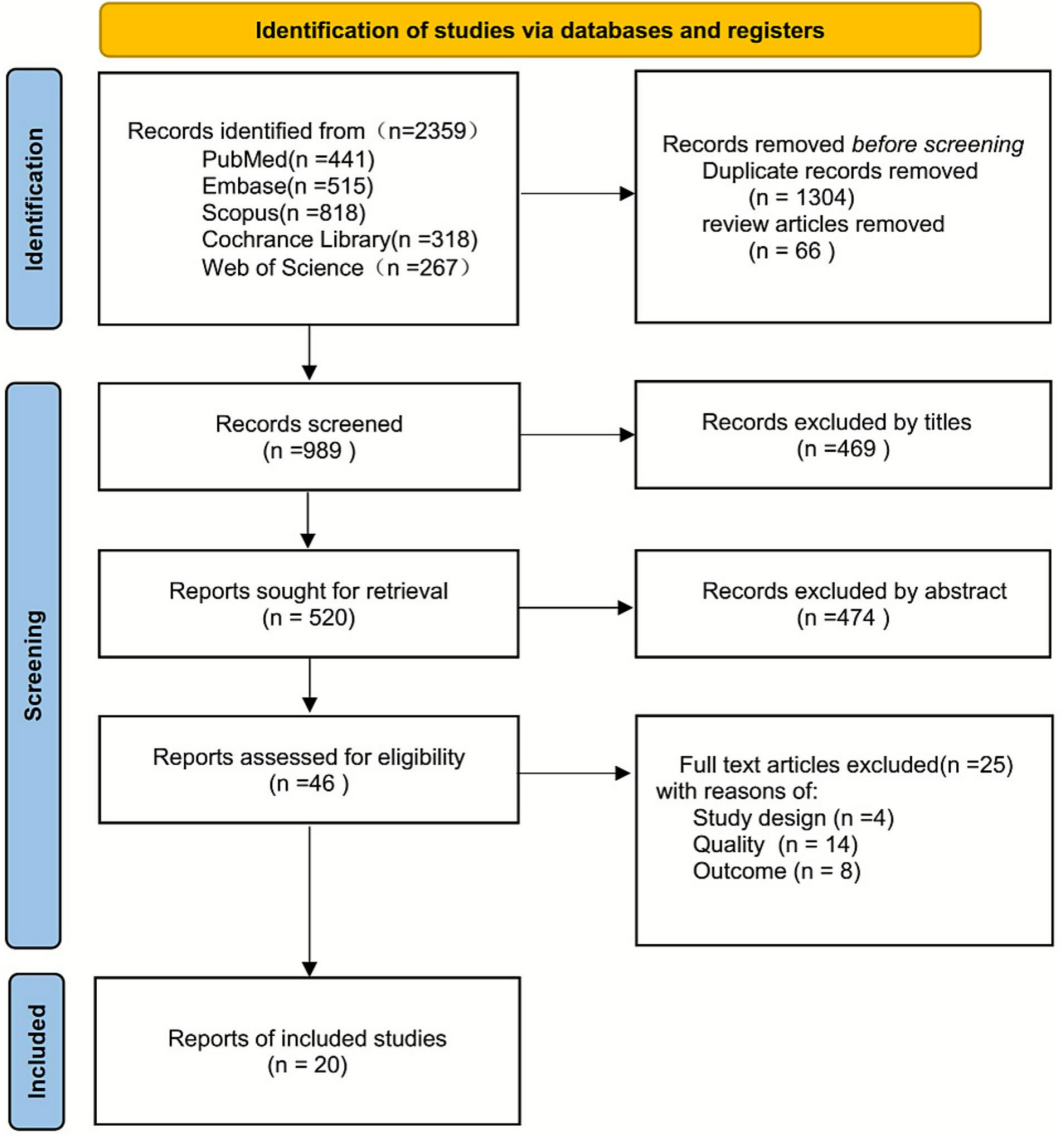
PRISMA flow diagram for study selection.

A total of 2,359 records were identified through searches of PubMed (*n* = 441), Embase (*n* = 515), Scopus (*n* = 818), Cochrane Library (*n* = 318), and Web of Science (*n* = 267). Based on the predefined inclusion and exclusion criteria, 20 studies (44–63) were ultimately included in the meta-analysis. The study selection process is illustrated in Figure 1.

### 3.2 Characteristics of included studies

All 20 studies (44–63) employed randomized crossover trial designs, encompassing a total of 226 participants, of whom 207 (91.6%) were male. Individual study sample sizes ranged from 6 to 20 participants, with a mean age between 20 and 48 years. Among the participants, 134 were well-trained cyclists (59.3%), 82 were recreational cyclists with some training experience (36.3%), and one study (10 participants, 4.4%) did not report training status. The performance outcomes assessed across studies included cycling completion time, mean power output, mean heart rate, and ratings of perceived exertion (RPE). Additional details are provided in Table 1.

**Table 1:** Basic characteristics of included studies.

Study (year)	Country	Sample (♂/♀)	Age	training level	Cycling protocol	Timing (min)	Doses (mg/kg)	From	Measures	Results
Backhouse 2011	UK	6	24 ± 1	Well-trained	90 min VO_2_ max 70%	60	6	Solution	③	③↑
Santos 2013	Brazil	8	32.6 ± 5.4	Well-trained	TT: 4KM	60	5	Capsule	①②③④	①↓②↑③↔④↔
Lee 2015	USA	15 (8/7)	37.3 ± 2.5	Well-trained	8mi*n* VO_2_ max 50%; 6 min 65% and 75%; TT:20.15 km	5	Total 70 mg	Tablets	①	①↓
Paton 2015	New Zealand	20 (10/10)	30 ± 10	Well-trained	TT:30 km	After 10 km	3–4	Gum	①②③④	①↓②↑③↔④↔
Bottoms 2014	UK	12	20.5 ± 0.7	Recreational	30 min	Every 6 min	Total 32 mg	Mouthwash	②③④	②↑③↔④↔
Quinlivan 2015	Australia	11	31.6 ± 6.1	Well-trained	60 min	90	3	Capsule	①②③④	①↓②↑③↑④↑
Kizzi 2016	UK	8	23 ± 2	Recreational	90 min VO_2_ max 70%; 5 × 6 s sprint	Before Each sprint	6	Mouthwash	②	②↑
Alvarenga 2019	Brazil	12	34.3 ± 6.2	Recreational	TT: 20 km	50	5	Capsule	①②④	①↓②↑④↓
Tomazini 2022	Brazil	11	33 ± 7	Well-trained	TT: 4 km	50	5	Capsule	①②④	①↓②↑④↓
Lei 2024	China	16	20 ± 2	Recreational	VO_2_ max 80%, 60 rpm until exhaustion	60	6	Capsule	①	①↓
Colmena 2024	Spain	11 (9/2)	22 ± 3	Recreational	TT: 13.9 km	60	3	Solution	①③④	①↓③↑④↔
John 2024	Australia	12	23 ± 4	Recreational	35 °C, 40%RH until exhaustion	60	5	Capsule	①	①↓
Santos 2011	Brazil	8	34.6 ± 5.0	Well-trained	TT: 4 km	60	5	Capsule	①②③	①↓②↑③↑
Hewitt 2012	Usa	10	28 ± 9	Na	20minVO_2_max 60%; TT: 20 km	60	6	Capsule	①②③④	①↓②↑③↔④↑
Spence 2013	Australia	10	30 ± 2	Well-trained	TT: 40 km	60	2.5	Capsule	①②③④	①↓②↑③↑④↔
Skinner 2013	Australia	14	31.0 ± 5.2	Well-trained	TT: 40 km	60	6	Capsule	①②	①↓②↑
Kilding 2012	New Zealand	10	24.2 ± 5.4	Well-trained	TT: 3 km	60	3	Capsule	①②③④	①↓②↑③↑④↓
Hodgson 2013	Uk	8	41 ± 7	Well-trained	30 mi*n* VO_2_max 55%; TT: 45 min	60	5	Capsule	①②	①↓②↑
Bortolotti 2014	Brazil	13	26 ± 10	Well-trained	TT:20 km	60	6	Capsule	①②③	①↓②↑③↔
Felippe 2018	Brazil	11	34 ± 4	Recreational	TT:4 km	75	5	Capsule	①	①↓

NA: Not available; TT: Time trial; ①: Completion time; ②: Mean power output; ③: Mean heart rate; ④: Ratings of perceived exertion.

### 3.3 Risk of bias assessment

The risk of bias in the included trials was primarily concentrated in the randomization process, outcome measurement, and selective reporting domains, each potentially contributing to some degree of bias. Overall, the proportion of studies rated as low risk, some concerns, and high risk was 35, 40, and 25%, respectively. Detailed results are presented in Supplementary material C.

### 3.4 Meta-analysis results

#### 3.4.1 Cycling completion time

Seventeen studies were included to examine the effect of caffeine ingestion on cycling completion time. Importantly, all 17 studies reported improvements in completion time following caffeine ingestion, consistently supporting its ergogenic benefit. The pooled effect size indicated that caffeine significantly reduced completion time (SMD = −0.36, 95% CI: −0.57 to −0.15, *p* < 0.01). Heterogeneity among studies was low, with no significant inconsistency observed (*Q* = 13.60, df = 19, *p* = 0.80, *I*^2^ = 0%).

#### 3.4.2 Mean power output

Data from 14 studies were analyzed to assess the effect of caffeine on mean power output during cycling. The pooled results showed that caffeine significantly increased mean power output (SMD = 0.29, 95% CI: 0.05 to 0.52, *p* < 0.05). Heterogeneity was low (*Q* = 4.90, df = 16, *p* = 1.00, *I*^2^ = 0%).

#### 3.4.3 Mean heart rate

Eleven studies were included to evaluate the effect of caffeine on mean heart rate during cycling. The pooled effect size was not statistically significant (SMD = 0.21, 95% CI: −0.06 to 0.48, *p* = 0.11). Heterogeneity was low, with good consistency among studies (*Q* = 10.99, df = 14, *p* = 0.69, *I*^2^ = 0%).

#### 3.4.4 Ratings of perceived exertion (RPE)

Ten studies examined the effect of caffeine on subjective fatigue (RPE) during exercise. The pooled effect size indicated a slight reduction in RPE in the caffeine group compared with the control, but the difference was not statistically significant (SMD = −0.03, 95% CI: −0.30 to 0.24, *p* = 0.82). Heterogeneity was low (Q = 10.38, df = 14, *p* = 0.73, I^2^ = 0%).

### 3.5 Publication bias

**Figure 2:**
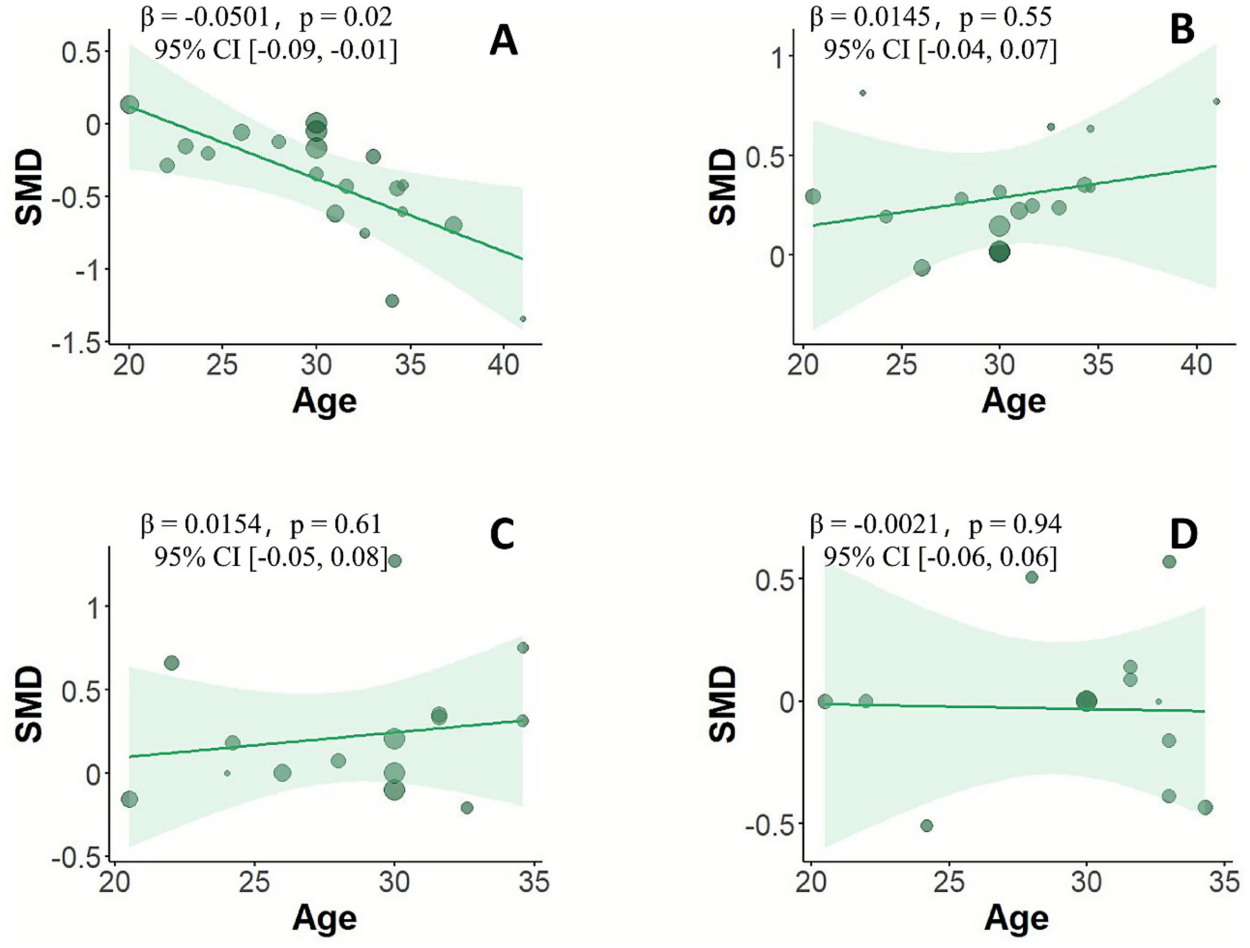
Meta-regression analysis of the effect of age on cycling performance.

**Figure 3:**
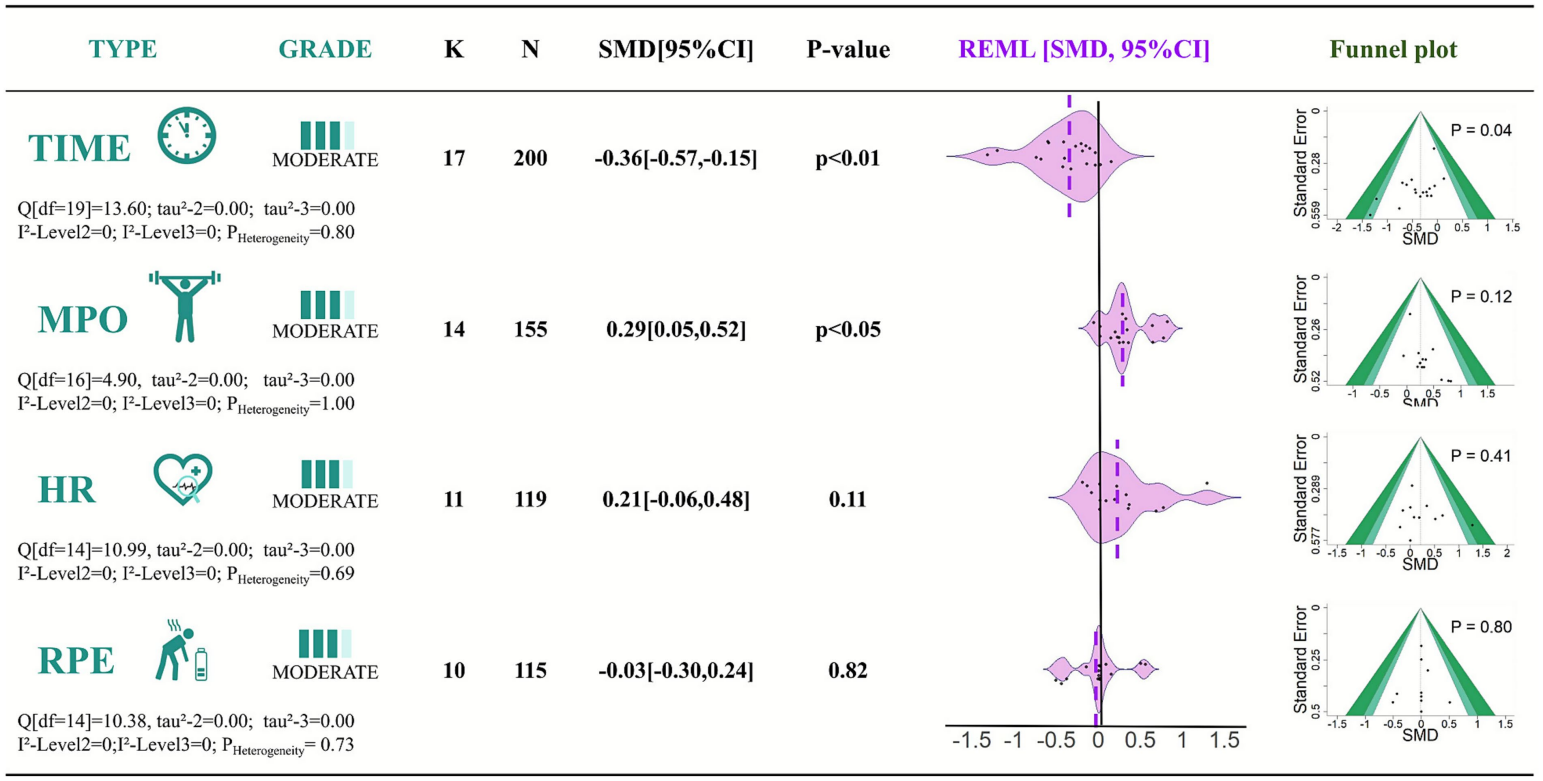
Effects of caffeine ingestion on cycling performance: time, power, HR, and RPE. *K*, number of studies; *N*, sample size; SMD, standardized mean difference; REML, restricted maximum likelihood: CI, confidence interval; TIME, completion time; MPO, mean power output; HR, mean heart rate; RPE, ratings of perceived exertion.

To assess potential publication bias, Egger’s regression test was conducted for the four outcome measures. The results indicated potential publication bias for cycling completion time (*p* < 0.05), with the corresponding funnel plot shown in Figure 2. No significant publication bias was observed for mean power output (*p* = 0.12), mean heart rate (*p* = 0.41), or ratings of perceived exertion (RPE) (*p* = 0.80), with funnel plots also presented in Figure 3.

### 3.6 Certainty of evidence

The certainty of evidence for the results of this systematic review is presented in Figure 3, with more detailed information available in Supplementary material E.

### 3.7 Sensitivity analysis

Leave-one-out sensitivity analyses demonstrated that exclusion of any single study did not alter the direction or statistical significance of the primary outcomes, indicating high robustness of the overall findings. Specifically: Completion time: SMD ranged from −0.33 to −0.45, all statistically significant (*p* < 0.01). Mean power output: *β* ranged from 0.22 to 0.30, approaching significance (*p* = 0.05–0.11). Mean heart rate: β ranged from 0.12 to 0.27, not significant (*p* > 0.11). RPE: β ranged from −0.04 to 0.08, not significant (*p* > 0.63).

### 3.8 Subgroup analyses

Caffeine dosage subgroup analysis (Table 2) revealed: Cycling completion time (TIME): Both ≤3 mg/kg (SMD = −0.42) and 4–6 mg/kg (SMD = −0.34) groups showed small reductions in completion time. Mean power output (MPO): 4–6 mg/kg group (SMD = 0.29) showed a small increase, whereas the ≤3 mg/kg group (SMD = 0.27) exhibited a smaller effect. Mean heart rate (HR): ≤3 mg/kg group (SMD = 0.41) showed a small increase, while the 4–6 mg/kg group (SMD = 0.07) exhibited a weaker effect. RPE: Both groups showed minimal effects (≤3 mg/kg: SMD = −0.06; 4–6 mg/kg: SMD = −0.004).

**Table 2:** Subgroup analyses based on caffeine dose and training level.

Subgroup	*K*	*N*	SMD [95%CI]	*P*	*I*^2^ [%]
TIME					
≤3 mg/kg	5	57	−0.42[−0.81,-0.02]	<0.05	0
4 ~ 6 mg/kg	12	143	−0.34[−0.59,-0.10]	<0.01	2.48
Well-trained	12	138	−0.38[−0.63,-0.13]	<0.01	0
Recreational	5	62	−0.32[−0.71,−0.06]	0.09	26.62
MPO					
≤3 mg/kg	4	43	0.27[−0.20,0.73]	0.24	0
4 ~ 6 mg/kg	10	112	0.29[0.02,0.57]	<0.05	0
Well-trained	11	123	0.25[−0.01,0.51]	0.06	0
Recreational	3	32	0.44[0.10,0.98]	<0.10	0
HR					
≤3 mg/kg	5	54	0.41[−0.01,0.82]	0.05	24.66
4 ~ 6 mg/kg	6	65	0.07[−0.29,0.42]	0.69	0
Well-trained	9	96	0.21[−0.09,0.51]	0.15	0
Recreational	2	23	0.23[−0.42,0.87]	0.46	46.53
RPE					
≤3 mg/kg	5	54	-0.06[−0.47,0.35]	0.75	0
4 ~ 6 mg/kg	5	61	−0.004[−0.37,0.36]	0.95	6.35
Well-trained	7	80	0.01[−0.30,0.33]	0.92	0
Recreational	3	35	−0.15[−0.67,0.37]	0.55	0

Training level subgroup analysis (Table 2) indicated: Completion time: Well-trained cyclists (SMD = −0.38) showed a small effect, slightly higher than recreational cyclists (SMD = −0.32). Mean power output (MPO): Both well-trained (SMD = 0.25) and recreational cyclists (SMD = 0.44) showed small improvements. Mean heart rate (HR): Both groups demonstrated small effects (well-trained: SMD = 0.21; recreational: SMD = 0.23). RPE: Effects were negligible (well-trained: SMD = 0.01; recreational: SMD = −0.15).

### 3.9 Meta-regression analysis

Meta-regression analyses indicated that age significantly moderated the effect of caffeine on cycling completion time (*β* = −0.0501, 95% CI: −0.09 to −0.01, *p* = 0.02), suggesting that the ergogenic effect of caffeine on completion time increases with age (Figure 2A). In contrast, age did not significantly moderate the effects of caffeine on mean power output, mean heart rate, or ratings of perceived exertion (RPE) (Figures 2B–D).

## 4 Discussion

Based on a systematic review and three-level mixed-effects meta-analysis, this study comprehensively examined the effects of acute caffeine ingestion on cycling performance, yielding the following main findings: acute caffeine intake significantly reduced cycling completion time and increased mean power output, indicating a clear ergogenic effect of caffeine on cycling performance. In contrast, caffeine had no significant impact on mean heart rate or ratings of perceived exertion (RPE). Importantly, the results also indicated a potential publication bias for cycling time-trial outcomes, suggesting that studies with null or unfavorable results may be underrepresented in the literature, which could lead to an overestimation of the true effect. Subgroup analyses further indicated that low-dose caffeine (≤3 mg/kg) produced a reduction in completion time that was comparable to, and may potentially be slightly greater than, the effect observed with moderate-to-high doses (4–6 mg/kg). Meta-regression analysis demonstrated that age significantly moderated the effect on completion time, with older athletes deriving greater benefits from caffeine ingestion.

Compared with the recent meta-analysis by Chen et al. (21), our findings show both consistency and notable differences. Similar to Chen et al. (21), we observed a significant ergogenic effect of caffeine on cycling time-trial performance and mean power output. However, in contrast to their report—where moderate doses (4–6 mg/kg) produced significant improvements and low doses (1–3 mg/kg) showed no clear effect—our results demonstrated significant benefits across all included studies, with both low and moderate doses producing small but meaningful reductions in completion time. Additionally, Chen et al. found no moderating effect of age, whereas our meta-regression indicated that older athletes may derive greater performance benefits. The observed improvements in completion time and power output further support caffeine’s ergogenic effects, and the low heterogeneity across included studies strengthens the reliability of these findings. The lack of significant effects on heart rate and RPE suggests that caffeine primarily enhances exercise efficiency and neuromuscular function, rather than directly affecting cardiovascular responses or subjective fatigue perception, which aligns with earlier reports (5–7).

Dose–response subgroup analyses indicated that low-dose caffeine may provide slightly superior reductions in completion time compared to moderate-to-high doses, supporting the notion that low doses can still elicit meaningful performance benefits (64, 65). Lower doses also minimize the risk of adverse effects and enhance long-term applicability, which has practical implications for athletic performance. Subgroup analyses based on training status showed that both well-trained and recreational cyclists benefited from caffeine supplementation, with slightly greater effects observed in well-trained athletes, suggesting that training level may modulate supplement efficacy.

The significant moderating effect of age is particularly noteworthy. Meta-regression results suggest that the ergogenic effect of caffeine on cycling completion time increases with age, possibly due to age-related declines in neuromuscular function, differences in caffeine metabolism, or heightened sensitivity. It is also possible that older participants in the included studies had longer training histories and higher fitness levels, contributing to greater benefits (66). These findings highlight the importance of considering individual age when designing nutrition supplementation strategies to achieve personalized, optimized interventions.

Strengths of this study include the inclusion of a substantial number of high-quality randomized crossover trials, the use of a three-level random-effects model to account for dependent effect sizes, which enhances statistical power and result robustness, and the detailed exploration of dose, training status, and age as moderating factors through subgroup and meta-regression analyses, providing deeper insights into the mechanisms underlying caffeine’s ergogenic effects.

However, several limitations should be acknowledged. First, the sample sizes in the included studies were relatively small, particularly for some subgroups and regression analyses, limiting statistical power. Future large-scale, high-quality trials are needed to confirm these findings. Second, factors such as caffeine administration form (capsules vs. beverages), exercise task type, and participants’ habitual caffeine intake were not fully controlled, potentially influencing outcomes. Additionally, most studies focused on short-duration, high-intensity cycling, so future research should extend to different endurance modalities and longer-duration performance assessments.

Nonetheless, this study still has several limitations. First, although the inclusion and exclusion criteria were defined according to the PICOS framework to ensure the specificity and focus of the research question, they may also introduce a certain degree of selection bias. For example, the exclusion of studies conducted under special experimental conditions—such as hypoxia, heat exposure, low-glycogen states, time-to-exhaustion (TTE) tests, and Wingate anaerobic sprint protocols—as well as studies involving combined supplementation protocols, may limit the generalizability of our findings to more complex or varied exercise contexts. Second, the overall sample size of the included studies remains relatively small, and the statistical power of certain subgroup analyses and multilevel meta-regression models may therefore be constrained. Moreover, 91.6% of the total sample (207 out of 226 participants) consisted of male cyclists, and no studies exclusively examined female cyclists. As a result, the results derived from the statistical analyses are more appropriately applied to male populations, and caution is required when attempting to generalize these findings to female cyclists. Third, variations in caffeine administration forms (e.g., capsules, beverages, mouth rinse), exercise task characteristics, and participants’ habitual caffeine intake were not fully controlled across studies, which may introduce additional heterogeneity into the results (67). Furthermore, most existing studies have focused on short-duration, high-intensity, or time-trial cycling tasks. Future research should extend to different endurance modalities, longer exercise durations, and populations with varied training backgrounds to more comprehensively assess the acute effects of caffeine.

Future investigations should integrate additional physiological markers and explore the mechanisms and individual variability in caffeine response, including genetic and sex-related differences, to facilitate the development of personalized supplementation strategies (11, 12). In practice, caffeine intake should be tailored based on athlete age, training status, and dose–response characteristics to maximize performance benefits while minimizing risk.

## 5 Conclusion

Caffeine ingestion significantly reduces cycling completion time and enhances mean power output, demonstrating its clear ergogenic effect on cycling performance. At the same time, current evidence shows that caffeine ingestion does not produce significant effects on heart rate or ratings of perceived exertion (RPE). Subgroup analysis showed that the ergogenic effect of low-dose caffeine was comparable to that of higher doses, indicating that lower doses can achieve comparable performance enhancement. Both well-trained and recreational athletes showed similar responses across outcome measures. Age was identified as an important moderator, with older athletes experiencing greater improvements in performance. Compared with previous studies, this review incorporated more high-quality trials and employed a three-level meta-analytic approach, enhancing the robustness and interpretability of the findings. Based on these results, it is recommended that athletes and coaches design caffeine supplementation strategies considering individual differences, including age, training status, and dietary habits, to optimize exercise performance outcomes.

## Data Availability

The original contributions presented in the study are included in the article/Supplementary material, further inquiries can be directed to the corresponding author/s.

## References

[1] Beck KL Thomson JS Swift RJ Von Hurst PR. Role of nutrition in performance enhancement and postexercise recovery. Open Access J Sports Med. (2015):259–67. 26316828 10.2147/OAJSM.S33605 PMC4540168

[2] Peeling P Castell LM Derave W de Hon O Burke LM. Sports foods and dietary supplements for optimal function and performance enhancement in track-and-field athletes. Int J Sport Nutr Exerc Metab. (2019) 29:198–209. doi: 10.1123/ijsnem.2018-0271, 30299192

[3] Burke LM. Caffeine and sports performance. Appl Physiol Nutr Metab. (2008) 33:1319–34. doi: 10.1139/H08-130, 19088794

[4] Guest NS VanDusseldorp TA Nelson MT Grgic J Schoenfeld BJ Jenkins ND . International society of sports nutrition position stand: caffeine and exercise performance. J Int Soc Sports Nutr. (2021) 18:1. doi: 10.1186/s12970-020-00383-4, 33388079 PMC7777221

[5] Davis J-K Green JM. Caffeine and anaerobic performance: ergogenic value and mechanisms of action. Sports Med. (2009) 39:813–32. doi: 10.2165/11317770-000000000-00000, 19757860

[6] Davis JM Zhao Z Stock HS Mehl KA Buggy J Hand GA. Central nervous system effects of caffeine and adenosine on fatigue. Am J Physiol Regul Integr Comp Physiol. (2003) 284. doi: 10.1152/ajpregu.00386.2002, 12399249

[7] Jones G. Caffeine and other sympathomimetic stimulants: modes of action and effects on sports performance. Essays Biochem. (2008) 44:109–24. 18384286 10.1042/BSE0440109

[8] Ganio MS Klau JF Casa DJ Armstrong LE Maresh CM. Effect of caffeine on sport-specific endurance performance: a systematic review. J Strength Cond Res. (2009) 23:315–24. doi: 10.1519/JSC.0b013e31818b979a, 19077738

[9] Jiménez-Alfageme R Garrone FP Rodriguez-Sanchez N Romero-García D Sospedra I Giménez-Monzó D . Nutritional intake and timing of marathon runners: influence of athlete’s characteristics and fueling practices on finishing time. Sports Med. (2025) 11:26. doi: 10.1186/s40798-024-00801-w, 40089940 PMC11911277

[10] Wang Z Qiu B Gao J Del Coso J. Effects of caffeine intake on endurance running performance and time to exhaustion: a systematic review and meta-analysis. Nutrients. (2022) 15:148. doi: 10.3390/nu15010148, 36615805 PMC9824573

[11] Lamberts RP Rietjens GJ Tijdink HH Noakes TD Lambert MI. Measuring submaximal performance parameters to monitor fatigue and predict cycling performance: a case study of a world-class cyclo-cross cyclist. Eur J Appl Physiol. (2010) 108:183–90. doi: 10.1007/s00421-009-1291-3, 19921241

[12] Nimmerichter A Eston R Bachl N Williams C. Effects of low and high cadence interval training on power output in flat and uphill cycling time-trials. Eur J Appl Physiol. (2012) 112:69–78. doi: 10.1007/s00421-011-1957-5, 21479957

[13] Nimmerichter A Eston RG Bachl N Williams C. Longitudinal monitoring of power output and heart rate profiles in elite cyclists. J Sports Sci. (2011) 29:831–9. 21500082 10.1080/02640414.2011.561869

[14] Anderson DE LeGrand SE McCart RD. Effect of caffeine on sprint cycling in experienced cyclists. J Strength Cond Res. (2018) 32:2221–6. doi: 10.1519/JSC.0000000000002685, 29912858

[15] Cole M Hopker JG Wiles JD Coleman DA. The effects of acute carbohydrate and caffeine feeding strategies on cycling efficiency. J Sports Sci. (2018) 36:817–23. doi: 10.1080/02640414.2017.1343956, 28644716

[16] Smirmaul BP de Moraes AC Angius L Marcora SM. Effects of caffeine on neuromuscular fatigue and performance during high-intensity cycling exercise in moderate hypoxia. Eur J Appl Physiol. (2017) 117:27–38. doi: 10.1007/s00421-016-3496-6, 27864638 PMC5306327

[17] McNaughton L Lovell RJ Siegler J Midgley AW Moore L Bentley DJ. The effects of caffeine ingestion on time trial cycling performance. Int J Sports Physiol Perform. (2008) 3:157–63. doi: 10.1123/ijspp.3.2.157, 19208924

[18] Paton CD Lowe T Irvine A. Caffeinated chewing gum increases repeated sprint performance and augments increases in testosterone in competitive cyclists. Eur J Appl Physiol. (2010) 110:1243–50. doi: 10.1007/s00421-010-1620-6, 20737165

[19] Wiles JD Coleman D Tegerdine M Swaine IL. The effects of caffeine ingestion on performance time, speed and power during a laboratory-based 1 km cycling time-trial. J Sports Sci. (2006) 24:1165–71. doi: 10.1080/02640410500457687 17035165

[20] Hunter AM St A Gibson C Collins M Lambert M Noakes TD. Caffeine ingestion does not alter performance during a 100-km cycling time-trial performance. Int J Sport Nutr Exerc Metab. (2002) 12:438–52. 12500987 10.1123/ijsnem.12.4.438

[21] Chen B Ding L Qin Q Lei T-H Girard O Cao Y. Effect of caffeine ingestion on time trial performance in cyclists: A systematic review and meta-analysis. J Int Soc Sports Nutr. (2024) 21:2363789. doi: 10.1080/15502783.2024.2363789, 38836626 PMC11155427

[22] Page MJ McKenzie JE Bossuyt PM Boutron I Hoffmann TC Mulrow CD . The PRISMA 2020 statement: an updated guideline for reporting systematic reviews. Int J Surg. (2021) 88:105906. doi: 10.1016/j.ijsu.2021.105906, 33789826

[23] Amir-Behghadami M Janati A. Population, intervention, comparison, outcomes and study (PICOS) design as a framework to formulate eligibility criteria in systematic reviews. Emerg Med J. (2020) 37:387. doi: 10.1136/emermed-2020-209567, 32253195

[24] Lucia A Pardo J Durántez A Hoyos J Chicharro JL. Physiological differences between professional and elite road cyclists. Int J Sports Med. (1998) 19:342–8. 9721058 10.1055/s-2007-971928

[25] McKinney J Velghe J Fee J Isserow S Drezner JA. Defining athletes and exercisers. Elsevier; 2019. p. 532–535. 10.1016/j.amjcard.2018.11.001 30503799

[26] Priego Quesada JI Kerr ZY Bertucci WM Carpes FP. The categorization of amateur cyclists as research participants: findings from an observational study. J Sports Sci. (2018) 36:2018–24. doi: 10.1080/02640414.2018.1432239, 29369014

[27] Sterne JAC Savović J Page MJ . RoB 2: a revised tool for assessing risk of bias in randomised trials. BMJ. (2019) 366:l4898 31462531 10.1136/bmj.l4898

[28] Desbrow B Biddulph C Devlin B Grant GD Anoopkumar-Dukie S Leveritt MD. The effects of different doses of caffeine on endurance cycling time trial performance. J Sports Sci. (2012) 30:115–20. doi: 10.1080/02640414.2011.632431, 22142020

[29] Filip-Stachnik A Wilk M Krzysztofik M Lulińska E Tufano JJ Zajac A . The effects of different doses of caffeine on maximal strength and strength-endurance in women habituated to caffeine. J Int Soc Sports Nutr. (2021) 18:25. doi: 10.1186/s12970-021-00421-9, 33781269 PMC8008648

[30] Guyatt G Oxman AD Akl EA Kunz R Vist G Brozek J . GRADE guidelines: 1. Introduction-GRADE evidence profiles and summary of findings tables. J Clin Epidemiol. (2011) 64:383–94. doi: 10.1016/j.jclinepi.2010.04.026, 21195583

[31] Viechtbauer W. Conducting meta-analyses in R with the metafor package. J Stat Softw. (2010) 36:1–48. doi: 10.18637/jss.v036.i03

[32] Pustejovsky JE Tipton E. Meta-analysis with robust variance estimation: expanding the range of working models. Prev Sci. (2022) 23:425–38. doi: 10.1007/s11121-021-01246-3, 33961175

[33] Polanin JR Hennessy EA Tanner-Smith EE. A review of meta-analysis packages in R. J Educ Behav Stat. (2017) 42:206–42.

[34] Cohen J. Statistical power analysis for the behavioral sciences routledge (2013).

[35] Assink M Wibbelink CJ. Fitting three-level meta-analytic models in R: a step-by-step tutorial. Quantitative Methods Psychol. (2016) 12:154–74. doi: 10.20982/tqmp.12.3.p154

[36] Harrer M Cuijpers P Furukawa TA Ebert DD. Doing meta-analysis with R: A hands-on guide. Boca Raton, FL: Chapman & Hall/CRC Press (2021).

[37] Kelley GA Kelley KS. Statistical models for meta-analysis: a brief tutorial. World J Methodol. (2012) 2:27. 25237614 10.5662/wjm.v2.i4.27 PMC4145560

[38] Tanner-Smith EE Tipton E Polanin JR. Handling complex meta-analytic data structures using robust variance estimates: a tutorial in R. J Dev Life-Course Criminol. (2016) 2:85–112. doi: 10.1007/s40865-016-0026-5

[39] Pustejovsky JE Tipton E. Small-sample methods for cluster-robust variance estimation and hypothesis testing in fixed effects models. J Bus Econ Stat. (2018) 36:672–83.

[40] Bowden J Tierney JF Copas AJ Burdett S. Quantifying, displaying and accounting for heterogeneity in the meta-analysis of RCTs using standard and generalised Q statistics. BMC Med Res Methodol. (2011) 11:41. doi: 10.1186/1471-2288-11-41, 21473747 PMC3102034

[41] Higgins JP Thompson SG. Quantifying heterogeneity in a meta-analysis. Stat Med. (2002) 21:1539–58. doi: 10.1002/sim.1186, 12111919

[42] Peters JL Sutton AJ Jones DR Abrams KR Rushton L. Contour-enhanced meta-analysis funnel plots help distinguish publication bias from other causes of asymmetry. J Clin Epidemiol. (2008) 61:991–6. doi: 10.1016/j.jclinepi.2007.11.010, 18538991

[43] Egger M Smith GD Schneider M Minder C. Bias in meta-analysis detected by a simple, graphical test. BMJ. (1997) 315:629–34. doi: 10.1136/bmj.315.7109.629, 9310563 PMC2127453

[44] Backhouse SH Biddle SJ Bishop NC Williams C. Caffeine ingestion, affect and perceived exertion during prolonged cycling. Appetite. (2011) 57:247–52. doi: 10.1016/j.appet.2011.05.304, 21605608

[45] Santos RA Kiss MAPDM Silva-Cavalcante MD Correia-Oliveira CR Bertuzzi R Bishop DJ . Caffeine alters anaerobic distribution and pacing during a 4000-m cycling time trial. PLoS One. (2013) 8:e75399. doi: 10.1371/journal.pone.0075399, 24058684 PMC3776790

[46] Lee J Kim H Solares G Kim K Ding Z Ivy J. Caffeinated nitric oxide-releasing lozenge improves cycling time trial performance. Int J Sports Med. (2015) 36:107–12. 25285468 10.1055/s-0034-1387762

[47] Paton C Costa V Guglielmo L. Effects of caffeine chewing gum on race performance and physiology in male and female cyclists. J Sports Sci. (2015) 33:1076–83. doi: 10.1080/02640414.2014.984752, 25517202

[48] Bottoms L Hurst H Scriven A Lynch F Bolton J Vercoe L . The effect of caffeine mouth rinse on self-paced cycling performance. Comp Exerc physiol. (2014) 10:239–45.

[49] Quinlivan A Irwin C Grant GD Anoopkumar-Dukie S Skinner T Leveritt M . The effects of red bull energy drink compared with caffeine on cycling time-trial performance. Int J Sports Physiol Perform. (2015) 10:897–901. doi: 10.1123/ijspp.2014-0481, 25710190

[50] Kizzi J Sum A Houston FE Hayes LD. Influence of a caffeine mouth rinse on sprint cycling following glycogen depletion. Eur J Sport Sci. (2016) 16:1087–94. doi: 10.1080/17461391.2016.1165739, 27686403

[51] Franco-Alvarenga PE Brietzke C Canestri R Goethel MF Hettinga F Santos TM . Caffeine improved cycling trial performance in mentally fatigued cyclists, regardless of alterations in prefrontal cortex activation. Physiol Behav. (2019) 204:41–8. doi: 10.1016/j.physbeh.2019.02.009, 30742838

[52] Tomazini F Santos-Mariano AC dos S Andrade VF Coelho DB Bertuzzi R Pereira G . Caffeine ingestion increases endurance performance of trained male cyclists when riding against a virtual opponent without altering muscle fatigue. Eur J Appl Physiol. (2022) 122:1915–28. doi: 10.1007/s00421-022-04969-5, 35612684

[53] Lei T-H Qin Q Girard O Mündel T Wang R Guo L . Caffeine intake enhances peak oxygen uptake and performance during high-intensity cycling exercise in moderate hypoxia. Eur J Appl Physiol. (2024) 124:537–49. doi: 10.1007/s00421-023-05295-0, 37608124

[54] Trujillo-Colmena D Fernández-Sánchez J Rodríguez-Castaño A Casado A Del Coso J. Effects of caffeinated coffee on cross-country cycling performance in recreational cyclists. Nutrients. (2024) 16:668. doi: 10.3390/nu16050668, 38474796 PMC10933887

[55] John K Kathuria S Peel J Page J Aitkenhead R Felstead A . Caffeine ingestion compromises thermoregulation and does not improve cycling time to exhaustion in the heat amongst males. Eur J Appl Physiol. (2024) 124:2489–502. doi: 10.1007/s00421-024-05460-z, 38568259 PMC11322244

[56] Santos PS Felippe LC Ferreira GA Learsi SK Couto PG Bertuzzi R . Caffeine increases peripheral fatigue in low-but not in high-performing cyclists. Appl Physiol Nutr Metab. (2020) 45:1208–15. doi: 10.1139/apnm-2019-0992, 32407654

[57] Acker-Hewitt TL Shafer BM Saunders MJ Goh Q Luden ND. Independent and combined effects of carbohydrate and caffeine ingestion on aerobic cycling performance in the fed state. Appl Physiol Nutr Metab. (2012) 37:276–83. doi: 10.1139/h11-160, 22436075

[58] Spence AL Sim M Landers G Peeling P. A comparison of caffeine versus pseudoephedrine on cycling time-trial performance. Int J Sport Nutr Exerc Metab. (2013) 23:507–12. doi: 10.1123/ijsnem.23.5.507, 23578950

[59] Skinner TL Jenkins DG Taaffe DR Leveritt MD Coombes JS. Coinciding exercise with peak serum caffeine does not improve cycling performance. J Sci Med Sport. (2013) 16:54–9. doi: 10.1016/j.jsams.2012.04.004, 22658588

[60] Kilding AE Overton C Gleave J. Effects of caffeine, sodium bicarbonate, and their combined ingestion on high-intensity cycling performance. Int J Sport Nutr Exerc Metab. (2012) 22:175–83. doi: 10.1123/ijsnem.22.3.175, 22693238

[61] Hodgson AB Randell RK Jeukendrup AE. The metabolic and performance effects of caffeine compared to coffee during endurance exercise. PLoS One. (2013) 8:e59561. doi: 10.1371/journal.pone.0059561, 23573201 PMC3616086

[62] Bortolotti H Altimari LR Vitor-Costa M Cyrino ES. Performance during a 20-km cycling time-trial after caffeine ingestion. J Int Soc Sports Nutr. (2014) 11:45. 25302056 10.1186/s12970-014-0045-8 PMC4190929

[63] Felippe LC Ferreira GA Learsi SK Boari D Bertuzzi R Lima-Silva AE. Caffeine increases both total work performed above critical power and peripheral fatigue during a 4-km cycling time trial. J Appl Physiol. (2018) 124:1491–501. doi: 10.1152/japplphysiol.00930.2017, 29470151

[64] Talanian JL Spriet LL. Low and moderate doses of caffeine late in exercise improve performance in trained cyclists. Appl Physiol Nutr Metab. (2016) 41:850–5. doi: 10.1139/apnm-2016-0053, 27426699

[65] Wang C Zhu Y Dong C Zhou Z Zheng X. Effects of various doses of caffeine ingestion on intermittent exercise performance and cognition. Brain Sci. (2020) 10:595. doi: 10.3390/brainsci10090595, 32872249 PMC7564618

[66] Khodadadi D Azimi F Eghbal Moghanlou A Gursoy R Demirli A Jalali P . Habitual caffeine consumption and training status affect the Ergogenicity of acute caffeine intake on exercise performance. Sports Health. (2025) 17:930–41. doi: 10.1177/19417381251315093, 39905628 PMC11795567

[67] Carvalho A Marticorena FM Grecco BH Barreto G Saunders B. Can I have my coffee and drink it? A systematic review and meta-analysis to determine whether habitual caffeine consumption affects the ergogenic effect of caffeine. Sports Med. 52. doi: 10.1007/s40279-022-01685-0, 35536449

