# Supplementary material for: Effect of caffeine ingestion on cycling performance: a systematic review and meta-analysis
Source: Front Nutr. 2026 Jan 12;12:1745472. doi: 10.3389/fnut.2025.1745472 (PMC12832238; doi:10.3389/fnut.2025.1745472)
Supplement: Supplementary file 1 [file Data_Sheet_1.pdf]

SUPPLEMENTARY MATERIAL

Table of Contents

Supplementary A: Search Strategy 2

Table 1. Database Search Details 2

Supplementary B: Forest Plot 3

Figure 1. Forest plot of completion time 3

Figure 2. Forest plot of mean power output 4

Figure 3. Forest plot of mean heart rate 5

Figure 4. Forest plot of ratings of perceived exertion 6

Supplementary C: Risk of Bias 7

Figure 5. Risk of bias assessment plot 7

Supplementary D: List of included studies 8

Supplementary E:GRADE levels of evidence 10

Table 2. GRADE Evidence Quality Assessment Table 10

Supplementary A: Search Strategy

Table 1. Database Search Details

Database	Search Strategy	Records
PubMed	(("caffeine"[Mesh] OR caffeine[tiab] OR coffee[tiab] OR "caffeine capsules"[tiab] OR "caffeinated gum"[tiab] OR "caffeine mouth rinse"[tiab] OR "caffeine liquid"[tiab] OR "caffeine pills"[tiab] OR "caffeine drinks"[tiab]) AND ("cycling"[Mesh] OR cycling[tiab] OR bicycling[tiab] OR "cycling performance"[tiab] OR "endurance cycling"[tiab]))	441
Embase	(('caffeine'/exp OR caffeine:ti,ab OR coffee:ti,ab OR 'caffeine capsules':ti,ab OR 'caffeinated gum':ti,ab OR 'caffeine mouth rinse':ti,ab OR 'caffeine liquid':ti,ab OR 'caffeine pills':ti,ab OR 'caffeine drinks':ti,ab) AND ('cycling'/exp OR cycling:ti,ab OR bicycling:ti,ab OR 'cycling performance':ti,ab OR 'endurance cycling':ti,ab))	515
Scopus	(TITLE-ABS-KEY(caffeine OR coffee OR "caffeine capsules" OR "caffeinated gum" OR "caffeine mouth rinse" OR "caffeine liquid" OR "caffeine pills" OR "caffeine drinks") AND TITLE-ABS-KEY(cycling OR bicycling OR "cycling performance" OR "endurance cycling")))	818
Cochrane Library	#1 (caffeine:ti,ab,kw OR coffee:ti,ab,kw OR "caffeine capsules":ti,ab,kw OR "caffeinated gum":ti,ab,kw OR "caffeine mouth rinse":ti,ab,kw OR "caffeine liquid":ti,ab,kw OR "caffeine pills":ti,ab,kw OR "caffeine drinks":ti,ab,kw) #2 (cycling:ti,ab,kw OR bicycling:ti,ab,kw OR "cycling performance":ti,ab,kw OR "endurance cycling":ti,ab,kw) #3 #1 AND #2	318
Web of Science	TS=(caffeine OR coffee OR "caffeine capsules" OR "caffeinated gum" OR "caffeine mouth rinse" OR "caffeine liquid" OR "caffeine pills" OR "caffeine drinks") AND TS=(cycling OR bicycling OR "cycling performance" OR "endurance cycling")	267

Supplementary B: Forest Plot

Study	COFF	Mean	SD	PLA	Mean	SD	REML (95% CI)	SMD [95% CI]
Felippe	11	396	5.00	11	403	6.00		−1.22 [−2.13, −0.31]
Bortolotti	13	2181	193.90	13	2191	157.60		−0.05 [−0.82, 0.71]
Hodgson	8	38.35	1.53	8	40.31	1.22		−1.34 [−2.42, −0.25]
Kilding	10	226.5	9.40	10	228.7	10.80		−0.21 [−1.09, 0.67]
Skinner	14	3475.7	97.20	14	3546.2	122.80		−0.62 [−1.38, 0.14]
Spence	10	4439	153.00	10	4494	153.00		−0.34 [−1.23, 0.54]
Hewitt	10	43.6	4.90	10	44.2	4.50		−0.12 [−1.00, 0.76]
Santos.3	8	402.5	16.10	8	412.7	15.60		−0.61 [−1.61, 0.39]
Santos.2	8	365.5	13.10	8	371	11.40		−0.42 [−1.41, 0.57]
John	12	28.5	8.30	12	29.9	8.80		−0.16 [−0.96, 0.64]
Colmena	11	41.17	6.18	11	43.2	7.35		−0.29 [−1.13, 0.55]
Lei	16	558	88.00	16	546	89.00		0.13 [−0.56, 0.83]
Tomazini	11	367.3	15.20	11	370.7	14.20		−0.22 [−1.06, 0.62]
Alvarenga	12	32.4	1.20	12	33	1.40		−0.44 [−1.25, 0.37]
Quinlivan	11	3757	278.00	11	3877	260.00		−0.43 [−1.27, 0.42]
Paton.3	20	1105	115.00	20	1126	131.00		−0.17 [−0.79, 0.45]
Paton.2	20	1120	128.00	20	1119	123.00		0.01 [−0.61, 0.63]
Paton.1	20	1049	129.00	20	1056	126.00		−0.05 [−0.67, 0.57]
Lee	15	2424	69.00	15	2477	78.00		−0.70 [−1.44, 0.04]
Santos.1	8	409.4	11.60	8	419.1	12.60		−0.76 [−1.77, 0.26]
Random−Effects Model								−0.36 [−0.57, −0.15]

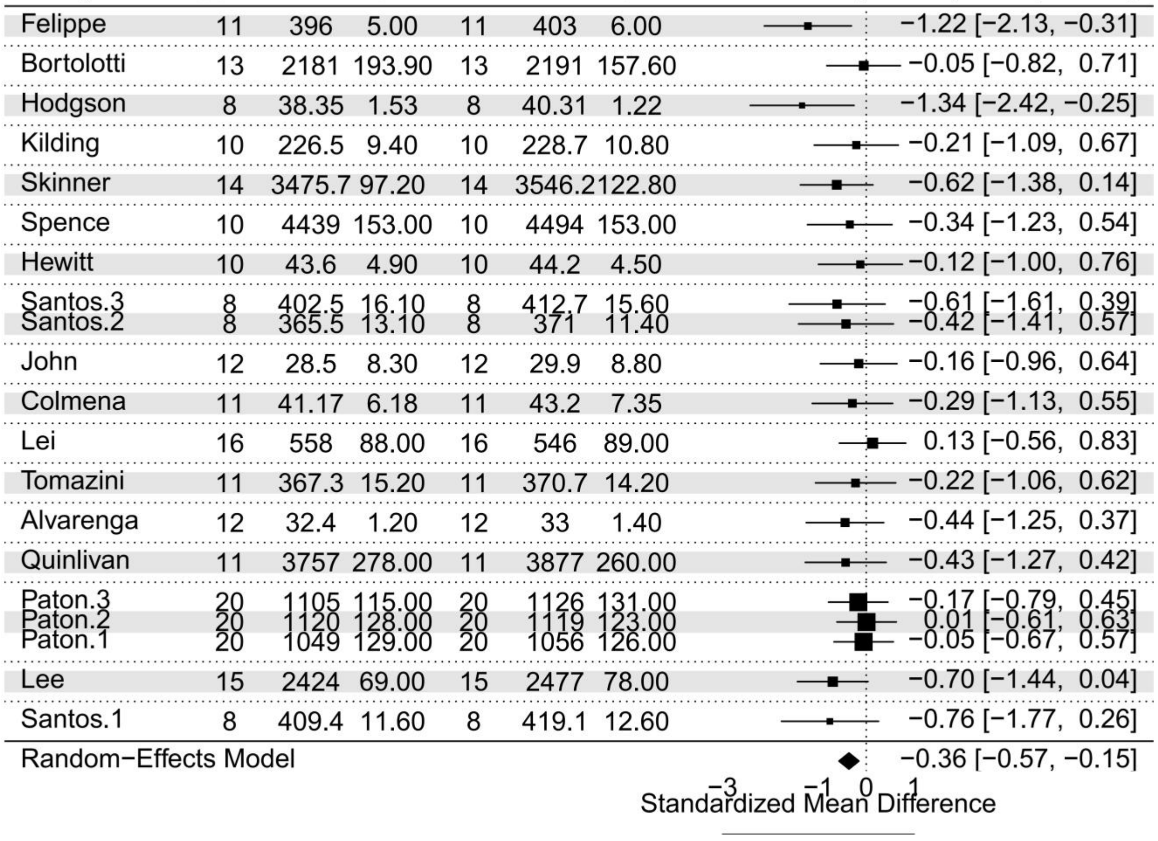

Figure 1. Forest plot of completion time

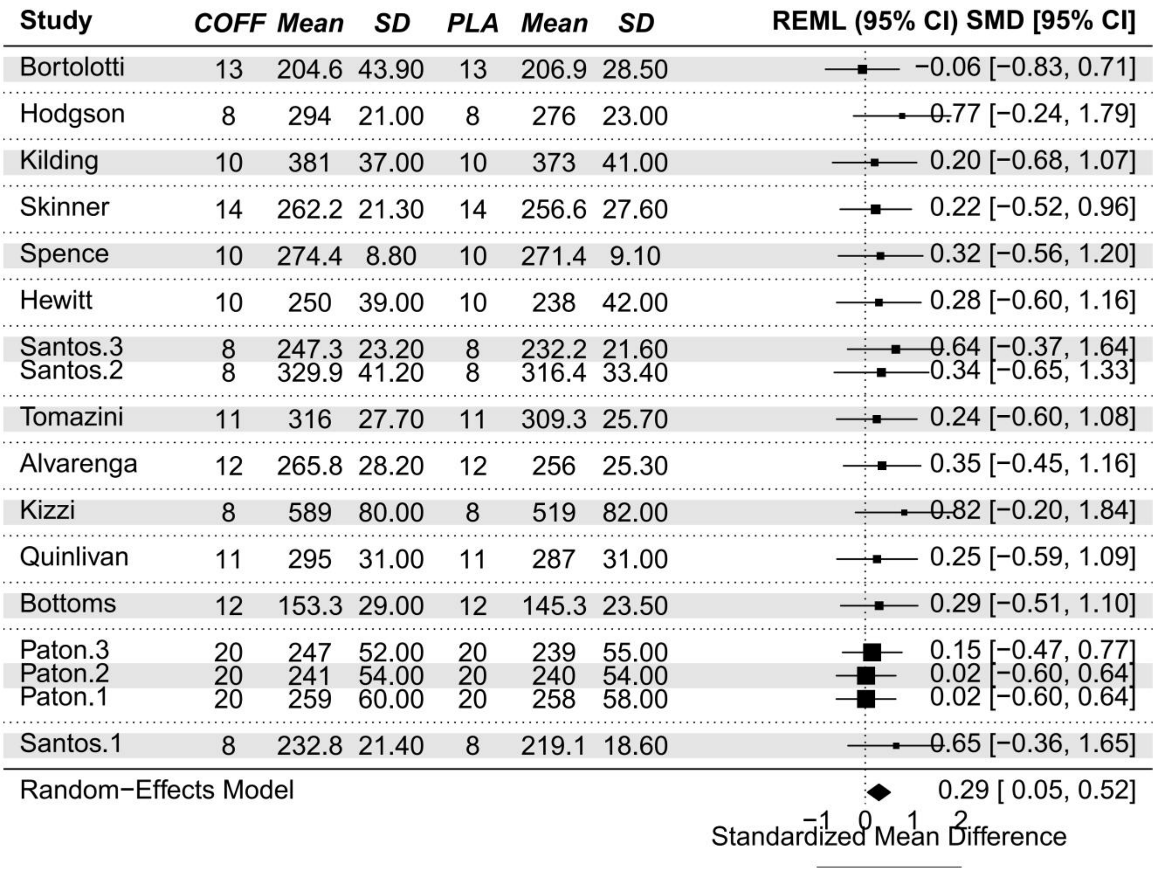

Figure 2. Forest plot of mean power output

Study	COFF	Mean	SD	PLA	Mean	SD	REML (95% CI) SMD [95% CI]
Bortolotti	13	171	8.00	13	171	9.90	0.00 [−0.77, 0.77]
Kilding	10	177	11.00	10	175	10.00	0.18 [−0.70, 1.06]
Spence	10	166	3.00	10	162	3.00	1.28 [0.32, 2.24]
Hewitt	10	179	11.00	10	178	14.00	0.08 [−0.80, 0.95]
Santos.3	8	162	7.00	8	156	8.00	0.75 [−0.26, 1.77]
Santos.2	8	162	5.00	8	160	7.00	0.31 [−0.68, 1.30]
Colmena	11	169	6.00	11	162	13.00	0.67 [−0.19, 1.52]
Quinlivan.2	11	176.7	9.50	11	172.9	11.40	0.35 [−0.49, 1.19]
Quinlivan.1	11	171.4	8.90	11	168.1	10.00	0.34 [−0.51, 1.18]
Bottoms	12	156	24.00	12	160	26.00	−0.15 [−0.96, 0.65]
Paton.3	20	166	9.00	20	164	10.00	0.21 [−0.42, 0.83]
Paton.2	20	163	9.00	20	163	9.00	0.00 [−0.62, 0.62]
Paton.1	20	158	9.00	20	159	10.00	−0.10 [−0.72, 0.52]
Santos.1	8	167	8.00	8	169	10.00	−0.21 [−1.19, 0.77]
Backhouse	6	159	3.00	6	159	3.00	0.00 [−1.13, 1.13]
Random−Effects Model							0.21 [−0.06, 0.48]

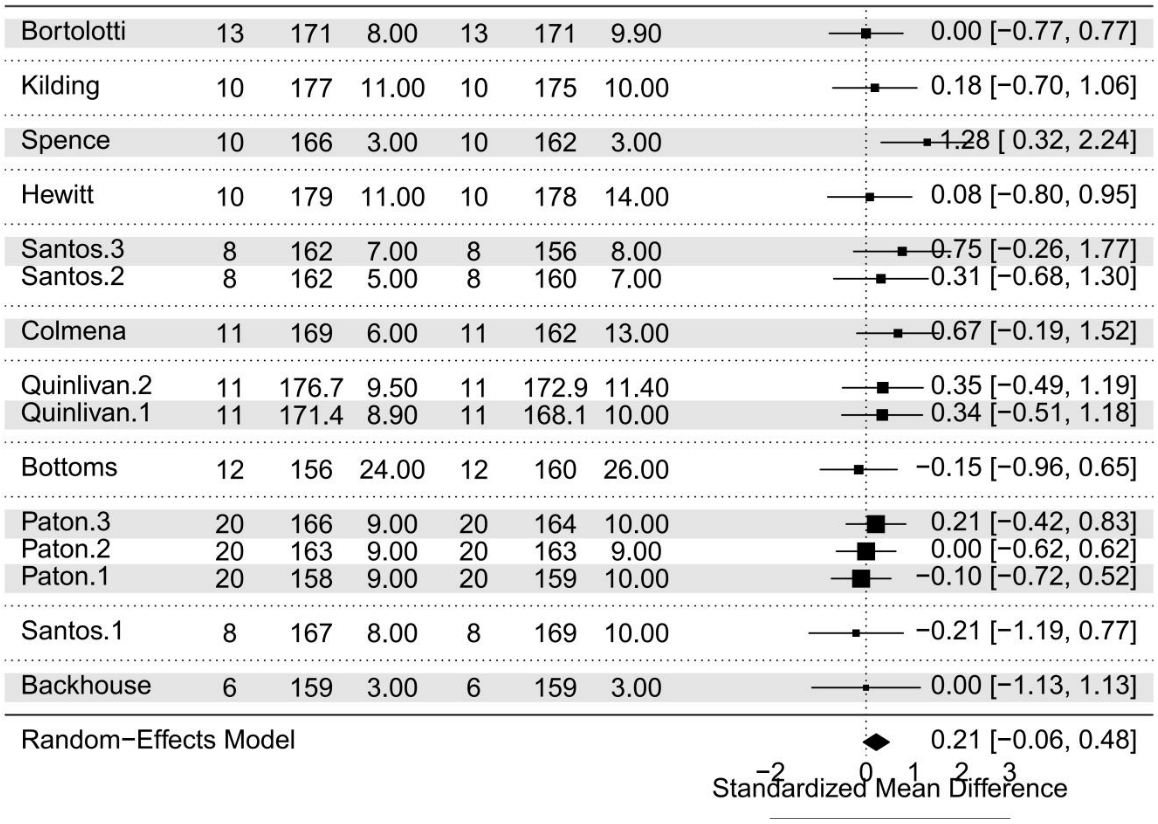

Figure 3. Forest plot of mean heart rate

Study	COFF	Mean	SD	PLA	Mean	SD	REML (95% CI) SMD [95% CI]
Kilding	10	3.9	0.80	10	4.3	0.70	−0.51 [−1.40, 0.38]
Spence	10	15	1.00	10	15	1.00	0.00 [−0.88, 0.88]
Hewitt	10	18.5	1.60	10	17.6	1.80	0.51 [−0.38, 1.40]
Colmena	11	16	1.00	11	16	2.00	0.00 [−0.84, 0.84]
Tomazini.3	11	18.4	1.90	11	17.3	1.80	0.57 [−0.28, 1.42]
Tomazini.2	11	15	2.70	11	15.4	2.00	−0.16 [−1.00, 0.68]
Tomazini.1	11	13.1	2.50	11	14	1.90	−0.39 [−1.23, 0.45]
Alvarenga	12	65.7	105.40	12	114.8	113.00	−0.43 [−1.24, 0.38]
Quinlivan.2	11	17.8	1.40	11	17.6	1.40	0.14 [−0.70, 0.97]
Quinlivan.1	11	15.3	1.00	11	15.2	1.20	0.09 [−0.75, 0.92]
Bottoms	12	13	2.00	12	13	1.00	0.00 [−0.80, 0.80]
Paton.3	20	17	1.00	20	17	1.00	0.00 [−0.62, 0.62]
Paton.2	20	16	1.00	20	16	1.00	0.00 [−0.62, 0.62]
Paton.1	20	15	1.00	20	15	1.00	0.00 [−0.62, 0.62]
Santos	8	14	2.00	8	14	1.00	0.00 [−0.98, 0.98]
Random−Effects Model							−0.03 [−0.30, 0.24]

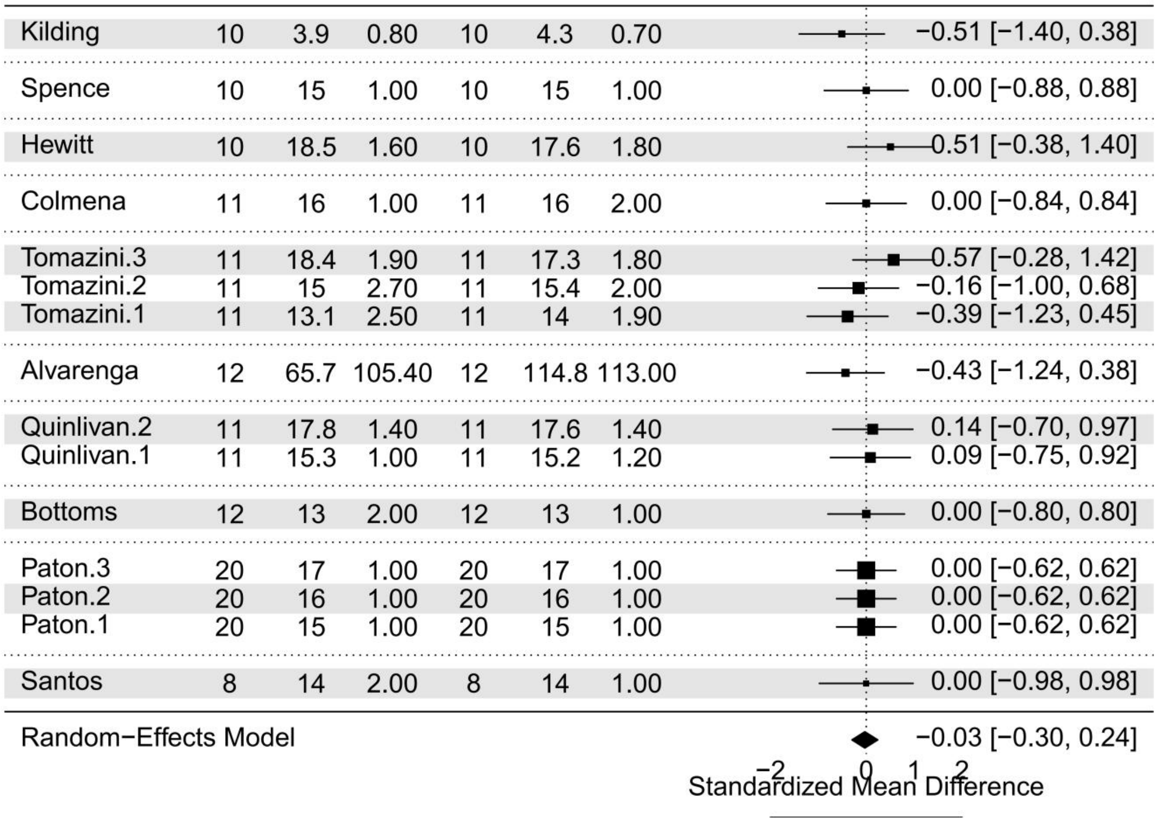

Figure 4. Forest plot of ratings of perceived exertion

Supplementary C: Risk of Bias

Study ID	D1	D2	D3	D4	D5	Overall
Backhouse2011	+	+	+	+	!	!
Santos2013	!	+	+	+	!	!
Lee2015	+	+	+	+	+	+
Paton2015	+	+	+	+	!	!
Bottoms2014	!	!	+	+	+	!
Quinlivan2015	+	+	+	+	+	+
Kizzi2016	!	!	+	+	+	!
Alvarenga2019	+	+	+	+	+	+
Tomazini2022	+	+	+	+	+	+
Lei2024	+	+	+	-	!	-
Colmena2024	+	+	+	+	!	!
John2024	+	+	+	-	!	-
Santos 2011	+	+	+	+	+	+
Hewitt2012	!	+	+	+	+	!
Spence2013	+	+	+	+	+	+
Skinner2013	+	+	+	-	!	-
Kilding2012	+	+	+	+	!	!

+ Low risk
! Some concerns
- High risk

D1 Randomisation process
D2 Deviations from the intended interventions
D3 Missing outcome data
D4 Measurement of the outcome
D5 Selection of the reported result

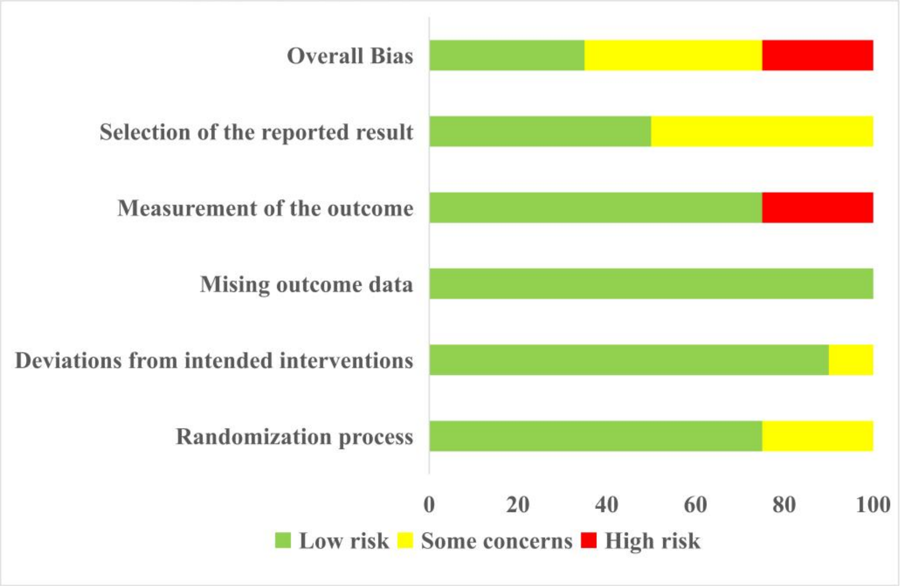

Figure 5. Risk of bias assessment plot

Supplementary D: List of included studies

1. Backhouse SH, Biddle SJ, Bishop NC, Williams C. Caffeine ingestion, affect and perceived exertion during prolonged cycling. Appetite. 2011;57(1):247-52.
2. Santos RdA, Kiss MAPDM, Silva-Cavalcante MD, Correia-Oliveira CR, Bertuzzi R, Bishop DJ, Lima-Silva AE. Caffeine alters anaerobic distribution and pacing during a 4000-m cycling time trial. PloS one. 2013;8(9):e75399.
3. Lee J, Kim H, Solares G, Kim K, Ding Z, Ivy J. Caffeinated nitric oxide-releasing lozenge improves cycling time trial performance. International journal of sports medicine. 2015;36(02):107-12.
4. Paton C, Costa V, Guglielmo L. Effects of caffeine chewing gum on race performance and physiology in male and female cyclists. Journal of Sports Sciences. 2015;33(10):1076-83.
5. Bottoms L, Hurst H, Scriven A, Lynch F, Bolton J, Vercoe L, et al. The effect of caffeine mouth rinse on self-paced cycling performance. Comp Exerc physiol. 2014;10(4):239-45.
6. Quinlivan A, Irwin C, Grant GD, Anoopkumar-Dukie S, Skinner T, Leveritt M, Desbrow B. The effects of Red Bull energy drink compared with caffeine on cycling time-trial performance. International journal of sports physiology and performance. 2015;10(7):897-901.
7. Kizzi J, Sum A, Houston FE, Hayes LD. Influence of a caffeine mouth rinse on sprint cycling following glycogen depletion. European journal of sport science. 2016;16(8):1087-94.
8. Franco-Alvarenga PE, Brietzke C, Canestri R, Goethel MF, Hettinga F, Santos TM, Pires FO. Caffeine improved cycling trial performance in mentally fatigued cyclists, regardless of alterations in prefrontal cortex activation. Physiology & behavior. 2019;204:41-8.
9. Tomazini F, Santos-Mariano AC, dos S. Andrade VF, Coelho DB, Bertuzzi R, Pereira G, et al. Caffeine ingestion increases endurance performance of trained male cyclists when riding against a virtual opponent without altering muscle fatigue. European Journal of Applied Physiology. 2022;122(8):1915-28.
10. Lei T-H, Qin Q, Girard O, Mündel T, Wang R, Guo L, Cao Y. Caffeine intake enhances peak oxygen uptake and performance during high-intensity cycling exercise in moderate hypoxia. European Journal of Applied Physiology. 2024;124(2):537-49.
11. Trujillo-Colmena D, Fernández-Sánchez J, Rodríguez-Castaño A, Casado A, Del Coso J. Effects of caffeinated coffee on cross-Country cycling performance in recreational cyclists. Nutrients. 2024;16(5):668.
12. John K, Kathuria S, Peel J, Page J, Aitkenhead R, Felstead A, et al. Caffeine ingestion compromises thermoregulation and does not improve cycling time to exhaustion in the heat amongst males. European Journal of Applied Physiology. 2024;124(8):2489-502.
13. Santos PS, Felippe LC, Ferreira GA, Learsi SK, Couto PG, Bertuzzi R, et al. Caffeine increases peripheral fatigue in low-but not in high-performing cyclists. Applied Physiology, Nutrition, and Metabolism. 2020;45(11):1208-15.
14. Acker-Hewitt TL, Shafer BM, Saunders MJ, Goh Q, Luden ND. Independent and combined effects of carbohydrate and caffeine ingestion on aerobic cycling performance

in the fed state. Applied Physiology, Nutrition, and Metabolism. 2012;37(2):276-83.
15. Spence AL, Sim M, Landers G, Peeling P. A comparison of caffeine versus pseudoephedrine on cycling time-trial performance. International journal of sport nutrition and exercise metabolism. 2013;23(5):507-12.
16. Skinner TL, Jenkins DG, Taaffe DR, Leveritt MD, Coombes JS. Coinciding exercise with peak serum caffeine does not improve cycling performance. Journal of science and medicine in sport. 2013;16(1):54-9.
17. Kilding AE, Overton C, Gleave J. Effects of caffeine, sodium bicarbonate, and their combined ingestion on high-intensity cycling performance. International journal of sport nutrition and exercise metabolism. 2012;22(3):175-83.
18. Hodgson AB, Randell RK, Jeukendrup AE. The metabolic and performance effects of caffeine compared to coffee during endurance exercise. PloS one. 2013;8(4):e59561.
19. Bortolotti H, Altimari LR, Vitor-Costa M, Cyrino ES. Performance during a 20-km cycling time-trial after caffeine ingestion. Journal of the International Society of Sports Nutrition. 2014;11(1):45.
20. Felippe LC, Ferreira GA, Learsi SK, Boari D, Bertuzzi R, Lima-Silva AE. Caffeine increases both total work performed above critical power and peripheral fatigue during a 4-km cycling time trial. Journal of Applied Physiology. 2018;124(6):1491-501.

Supplementary E:GRADE levels of evidence

Table 2. GRADE Evidence Quality Assessment Table

CAF vs PLA	N（K）	Certainty of Evidence Assessment					SMD [95%CI]	Certainty of Evidence
		Risk of Bias	Inconsistency	Indirectness	Imprecision	Publication Bias		
TIME	210（18RCT）	Not serious	Not serious	Not serious	Not serious	Serious	-0.35 [-0.55,-0.15]	Moderate
MPO	165（15RCT）	Not serious	Not serious	Not serious	Not serious	Serious	0.36 [0.13,0.59]	Moderate
HR	119（11RCT）	Not serious	Not serious	Not serious	Serious	Not serious	0.21 [-0.06,0.48]	Moderate
RPE	115（10RCT）	Not serious	Not serious	Not serious	Serious	Not serious	-0.03 [-0.30,0.24]	Moderate

*GRADE Quality of Evidence and Strength of Recommendations:
High: The panel has high confidence in the estimated effect.
Moderate: The panel has moderate confidence in the estimated effect.
Low: The panel has limited confidence in the estimated effect.
Very low: The panel has very little confidence in the estimated effect.
